# The County Council and an Ambulance Service

**Published:** 1913-12-20

**Authors:** 


### THE COUNTY COUNCIL AND AN AMBULANCE SERVICE.

### The Report and Debate of Tuesday's Meeting.

The London County Council at its meeting on Tuesday, December 16, had under consideration important reports from its General Purposes Committee with regard to the provision of an ambulance service for the metropolis. It was this committee which had considered the scheme submitted by the Metropolitan Asylums Board, already published in The Hospital, and the committee regretted that they were unable to advise the Council to adopt it.

The General Purposes Committee in their report state that without attempting detailed criticism of the scheme there are two primary objections to it. First they are advised that the scheme put forward is not of such a nature as was contemplated by the Council, or as is authorised by the Metropolitan Ambulances Act of 1909, or the amendment to it which the Council obtained in its General Powers Act this year enabling the use of ambulances owned by other public authorities in London, and they are further advised that the Asylums Board would be acting *ultra vires* in giving effect thereto.

The committee state that they at once proceeded to consider possible alternative schemes for securing the establishment of a service. They recognise the necessity for immediate action, and a special sub-committee has been appointed to prepare a scheme.

### The Sub-Committee's Urgency Report.

The General Purposes Committee also submitted an urgency report from the sub-committee which had been appointed to prepare an ambulance scheme. This report contained an interesting outline of the scheme it is proposed to adopt.

The sub-committee stated that the short time at their disposal had not, of course, admitted of the preparation of any detailed scheme. but they could give a general indication of the lines upon which they were proceeding. They also explained that their scheme would be based on existing facilities. They had given consideration to the question of the co-ordination of existing appliances, and on this point they would state that these should be used only as supplemental to the service to be established by the Council. As an illustration they would refer to the possibility of using, as a supplement, such motor-ambulances as might in the future be adopted for the education service, such use being, of course, secondary, and in no way interfering with the primary objects for which such vehicles were provided. They proposed to include in their scheme the motor-ambulance at Hampstead General Hospital, which was already available, by arrangement with the Council, for street accident cases.

In framing the outlines of a scheme their first consideration was directed to the methods to be adopted for summoning the ambulances when provided. They were proceeding upon the basis of the Post-Office telephone system without the installation of an expensive system of call-boxes—calls being made not direct to ambulance stations, but to central stations, from which calls might be transmitted to ambulance stations. The next question was the provision of the ambulance stations, and here they had turned their attention to the suitability of buildings and sites already in the possession of the Council. They were proceeding with the idea of establishing, provisionally, stations equipped with motor-ambulances in the west, north, east, south-east, south, and south-west districts with a station in Central London. In addition to motor-ambulances they contemplated the provision of efficient hand-ambulances or litters. They had considered what department should be entrusted with the management of the service. In view of the resemblance between such a service and that of the fire-brigade they were proceeding to secure that the service should be administered not as an adjunct to the fire-brigade service, but under the supervision of its chief officer.

They hoped to submit a detailed scheme with estimates of cost when the Council reassembled after the Christmas recess.

### The Debate on the Reports.

Major Ernest Gray, Chairman of the General Purposes Committee, who submitted the reports, stated that the Asylums Board refused to consider the question of dual control. Their solicitor had advised the Council that a large portion of the scheme would be *ultra vires*, and that the Board would not have the power to give effect to it. The scheme contained no single existing motor-ambulance, the suggestion being that the whole of the ambulances should be provided at the expense of the Council, and that was a fatal objection. They might still be able to induce the Asylums Board to co-operate with them without becoming their agents and taking over complete control.

Mr. Geoffrey Drage thought that the Asylums Board had not been very fairly treated in the matter. He trusted that it would be made clear to the Board that the door was open to future negotiations. He did not think the Council's scheme would be as efficient as that submitted by the Asylums Board. The latter was complete and could be put into effect at once, whereas the Council's scheme would take some time.

Mr. L. W. S. Rostron hoped that the door would not be barred against further negotiations with the Asylums Board, but Sir John Benn hoped that there would be no more "fooling about" with the Asylums Board, and that the Council would now go on with the work.

The recommendation not to adopt the Asylums Board's scheme was adopted without dissent.

The General Purposes Committee further recommended that they should submit a detailed scheme for the establishment and maintenance by the Council, in co-operation, where possible, with other public authorities within the county, of an ambulance service for London, which was agreed to.

